# Editorial: Advances in understanding the pathogenesis of and designing therapies for connective tissue disease-associated interstitial lung disease

**DOI:** 10.3389/fimmu.2023.1295647

**Published:** 2023-10-16

**Authors:** Lihua Duan, Yan Yang, Xiaoxia Zhu

**Affiliations:** ^1^ Department of Rheumatology and Clinical Immunology, Jiangxi Provincial People’s Hospital, The First Affiliated Hospital of Nanchang Medical College, Nanchang, China; ^2^ Department of Thoracic/Head and Neck Medical Oncology, University of Texas MD Anderson Cancer Center, Houston, TX, United States; ^3^ Division of Rheumatology, Huashan Hospital, Fudan University, Shanghai, China

**Keywords:** interstitial lung disease, connective tissue disease, fibrosis, autoimmunity, rheumatoid arthritis

Interstitial lung disease (ILD) is also the most common manifestation of connective tissue diseases (CTD), including rheumatoid arthritis (RA), systemic sclerosis (SSc), and polymyositis (PM) or dermatomyositis (DM). Decreased lung function and worsening respiratory symptoms are common factors of death in CTD-ILD patients, which are caused by progressive lung fibrosis. Although multiple clinical trials have shown that nintedanib and pirfenidone are safe and effective in the treatment of CTD-ILD, patients with CTD-ILD who receive glucocorticoids or/and immunosuppressants are more likely to suffer from pulmonary infections. Li et al. construct a nomogram based on the five variables (fever, cyanosis, blood urea nitrogen, ganciclovir treatment, and anti-pseudomonas treatment) for predicting 90-day mortality in CTD-ILD with pneumonia patients using machine learning. Recent studies have proposed some mechanisms involved in the occurrence and development of ILD, such as pro-inflammatory and/or pro-fibrotic immune cells and cytokines, impaired endothelial cells, and fibroblast dysfunction (1). This Research Topic exhibits a number of clinical research articles on the topic of advances in the pathogenesis of and therapies for CTD-ILD.

RA is a chronic systemic autoimmune disease that can cause progressive articular and extra-articular damage, and ILD is the most common extra-articular manifestation of RA and its prevalence was reported to be up to 60% during the disease course (2). Importantly, ILD is the second leading cause of death in RA, after cardiovascular disease (3). Non-enzymatic chitinase-3 like-protein-1 (CHI3L1) belongs to the glycoside hydrolase family 18 and it plays a vital role in inflammation, tissue repair, and remodeling responses. In this Research Topic, Yu et al. analyze the association between CHI3L1 and the presence of RA-ILD, and they find that serum CHI3L1 levels were significantly higher in RA-ILD patients and had the ability to assist in the diagnosis of ILD in RA patients. Therefore, exploring new markers will facilitate early diagnosis of the disease. Iguratimod (IGU), which has novel anti-inflammatory and immunomodulatory properties, was developed as a new antirheumatic drug and was approved for active RA in China and Japan. IGU inhibits the production of immunoglobulins, autoantibodies, and various inflammatory cytokines (IL-1β, IL-17, and TNF-α), and osteoclastogenesis. Recent studies have shown that IGU also has anti-fibrotic effects (4). In this topic, Wang et al. demonstrate that IGU combined with tofacitinib can relieve both RA and RA with usual interstitial pneumonia (UIP) and is better than csDMARDs, with a higher response rate in RA-UIP patients. These results indicate that IGU not only suppresses the disease activity but also subsequently blocks subsequent fibrosis progression.

The ILD prevalence in DM/PM patients ranges from 23.1 to 65%, and it is worth noting that anti-MDA5+ DM patients have a higher incidence of ILD (90–95%) (5). Particularly, anti-MDA5+ DM frequently develops rapidly progressive interstitial lung disease (RP-ILD) (50–80%) with poor 90-day survival, especially in Asian patients (6, 7). Because the expression of type I interferon (IFN-I) stimulated genes is significantly elevated in the skin, lungs, and peripheral blood of DM patients, the involvement of IFN-I has been proposed in the pathogenesis of anti-MDA5+ DM. Qian et al. evaluate the expression of IFN-stimulated genes to determine the IFN-I score and find that the IFN-I score is a valuable tool to monitor disease activity and predict mortality in patients with anti-MDA5+ DM., Tofacitinib has consistently been used in the treatment of anti-MDA5+ DM-ILD by inhibiting the IFN-I signaling pathway (6, 7). However, the IFN-I signaling pathway was not activated in all the anti-MDA5 patients with ILD. Therefore, it is very important to evaluate the clinical phenotype and IFN-I scores in patients with anti-MDA5+DM, which will help implement early targeted and individualized therapy for high-risk patients. There are similarities between COVID-19 and anti-MDA5+ DM in terms of clinical features, pulmonary involvement, and immune mechanisms, especially IFN-I (8). In addition, Smail et al. observe that COVID-19 patients with high IL-10, IL-23, and TNFα on admission are more likely to experience a severe form of the disease, such as respiratory failure, septic shock, or multiple organ failure.

Collectively, the original research in this Research Topic covers a series of important aspects in the field of disease markers for and clinical management of CTD-ILD which may provide new insights into its diagnosis and treatment.

## Author contributions

LD: Writing – original draft, Writing – review & editing. YY: Writing – original draft, Writing – review & editing. XZ: Writing – original draft, Writing – review & editing.

## References

[1] Spagnolo P Distler O Ryerson CJ Tzouvelekis A Lee JS Bonella F. Mechanisms of progressive fibrosis in connective tissue disease (ctd)-associated interstitial lung diseases (ilds). Ann Rheumatol Dis (2021) 80:143–50. doi: 10.1136/annrheumdis-2020-217230 PMC7815631 33037004

[2] Norton S Koduri G Nikiphorou E Dixey J Williams P Young A. A study of baseline prevalence and cumulative incidence of comorbidity and extra-articular manifestations in ra and their impact on outcome. RHEUMATOLOGY (2013) 52:99–110. doi: 10.1093/rheumatology/kes262 23086517

[3] Finckh A Gilbert B Hodkinson B Bae SC Thomas R Deane KD. Global epidemiology of rheumatoid arthritis. Nat Rev Rheumatol (2022) 18:591–602. doi: 10.1038/s41584-022-00827-y 36068354

[4] Han Q Zheng Z Liang Q Fu X Yang F Xie R. Iguratimod reduces b-cell secretion of immunoglobulin to play a protective role in interstitial lung disease. Int Immunopharmacol (2021) 97:107596. doi: 10.1016/j.intimp.2021.107596 33892300

[5] Lundberg IE Fujimoto M Vencovsky J Aggarwal R Holmqvist M Christopher-Stine L. Idiopathic inflammatory myopathies. Nat Rev Dis Primers (2021) 7:86. doi: 10.1038/s41572-021-00321-x 34857798

[6] Kurasawa K Arai S Namiki Y Tanaka A Takamura Y Owada T. Tofacitinib for refractory interstitial lung diseases in anti-melanoma differentiation-associated 5 gene antibody-positive dermatomyositis. RHEUMATOLOGY (2018) 57:2114–9. doi: 10.1093/rheumatology/key188 30060040

[7] Chen Z Wang X Ye S. Tofacitinib in amyopathic dermatomyositis–associated interstitial lung disease. N Engl J Med (2019) 381:291–3. doi: 10.1056/NEJMc1900045 31314977

[8] Giannini M Ohana M Nespola B. Similarities between covid-19 and anti-mda5 syndrome: what can we learn for better care? Eur. Respir J (2020) 56:2001618. doi: 10.1183/13993003.01618-2020 PMC7338399 32631836

